# Rapid Generation of Recombinant Flaviviruses Using Circular Polymerase Extension Reaction

**DOI:** 10.3390/vaccines11071250

**Published:** 2023-07-17

**Authors:** Hao-Long Dong, Mei-Juan He, Qing-Yang Wang, Jia-Zhen Cui, Zhi-Li Chen, Xiang-Hua Xiong, Lian-Cheng Zhang, Hao Cheng, Guo-Qing Xiong, Ao Hu, Yuan-Yuan Lu, Chun-Lin Cheng, Zhi-Xin Meng, Chen Zhu, Guang Zhao, Gang Liu, Hui-Peng Chen

**Affiliations:** 1 Academy of Military Medical Sciences, Beijing 100071, China; haolong_dong@163.com (H.-L.D.); chenzl0708@163.com (Z.-L.C.); xxianghuabj@163.com (X.-H.X.);; 2 Institutes of Physical Science and Information Technology, Anhui University, Hefei 230000, China; 3 School of Life Science, Hebei University, Baoding 071000, China

**Keywords:** flavivirus, CPER, reverse genetic, vaccine, reporter virus

## Abstract

The genus *Flavivirus* is a group of arthropod-borne single-stranded RNA viruses, which includes important human and animal pathogens such as Japanese encephalitis virus (JEV), Zika virus (ZIKV), Dengue virus (DENV), yellow fever virus (YFV), West Nile virus (WNV), and Tick-borne encephalitis virus (TBEV). Reverse genetics has been a useful tool for understanding biological properties and the pathogenesis of flaviviruses. However, the conventional construction of full-length infectious clones for flavivirus is time-consuming and difficult due to the toxicity of the flavivirus genome to *E. coli*. Herein, we applied a simple, rapid, and bacterium-free circular polymerase extension reaction (CPER) method to synthesize recombinant flaviviruses in vertebrate cells as well as insect cells. We started with the de novo synthesis of the JEV vaccine strain SA-14-14-2 in Vero cells using CPER, and then modified the CPER method to recover insect-specific flaviviruses (ISFs) in mosquito C6/36 cells. Chimeric Zika virus (ChinZIKV) based on the Chaoyang virus (CYV) backbone and the Culex flavivirus reporter virus expressing green fluorescent protein (CxFV-GFP) were subsequently rescued in C6/36 cells. CPER is a simple method for the rapid generation of flaviviruses and other potential RNA viruses. A CPER-based recovery system for flaviviruses of different host ranges was established, which would facilitate the development of countermeasures against flavivirus outbreaks in the future.

## 1. Introduction

Virus outbreaks, especially zoonotic RNA viruses, regularly arise around the globe. The genus *flavivirus* contains a group of medically important viral agents such as DENV, ZIKV, JEV, TBEV, and WNV, transmitted between humans and vertebrates by bites of mosquitoes and ticks. These flaviviruses continue to pose a threat to public health, causing millions of infections and tens of thousands of deaths annually. Pathogenic flaviviruses are emerging and reemerging around the globe as well as expanding geographically due to increasing urbanization and globalization as well as climate change [1]. ZIKV outbreak was declared a Public Health Emergency of International Concern for its association with newborn microcephaly in 2016 [2]. Despite the widespread use of effective vaccines, YFV kept remerging in Africa and South America and caused millions of infections and tens of thousands of deaths in an outbreak in 2018 [3]. WNV kept expanding its geographical spectrum and caused an outbreak after its introduction to North America in 1999 [4]. In addition, more than a thousand cases of WNV infection have been reported during the last decade in Canada [5]. Zoonotic viruses are hard to control partly because of their persistence in wildlife reservoir hosts.

Members of the genus *flavivirus* have a plus-strand RNA genome of approximately 11 kb. Flavivirus genome encodes a single open reading frame (ORF), flanked by 5′ and 3′ untranslated regions (UTR), with a cap at the start of the 5′UTR and no polyA tail at the end of the 3′UTR [6]. Both the ORF and the UTRs contain complex RNA structures essential to virus replication [7,8,9]. The ORF encodes a polyprotein processed by viral and cellular proteases into three structural proteins, designated as capsid (C), premembrane (prM), and envelope (E), and seven non-structural proteins (NS1, NS2A, NS2B, NS3, NS4A, NS4B, NS5). According to host range, flaviviruses can be divided into classical insect-specific flaviviruses (cISFs), dual-host affiliated insect-specific flaviviruses (dISFs), mosquito/vertebrate flaviviruses, tick/vertebrate flaviviruses, and no known-vector flaviviruses [10]. Both the cISFs and the dISFs do not infect vertebrates and transmit vertically and/or horizontally between mosquitoes. However, their inability to infect vertebrates may have evolved separately, as dISFs possibly lost the ability to infect vertebrates whereas cISFs may never have extended their host range to vertebrates [10].

Currently, there are only commercial human vaccines for YFV, DENV, JEV, and no approved antivirals are available for flaviviruses [11]. However, YFV vaccine strain 17D has been reported to cause vaccine-associated viscerotropic disease and neurotropic disease in rare vaccinees [12,13,14,15]. The mechanism behind these severe adverse effects remains partly elusive. DENV vaccine development efforts have been hampered by antibody-dependent enhancement (ADE) [16]. JEV is a clinically important arthropod-borne zoonotic flavivirus circulating between humans and vertebrate hosts via mosquito bites. Domestic pigs are the main source of JEV in a zoonotic cycle for their ability to amplify JEV to high titers and their close proximity to humans [17,18]. JEV poses a huge threat to domestic pig farms and causes economic damage [18]. JEV infection in humans causes clinical presentations ranging from febrile illness to viral encephalitis. JEV vaccines have been proven successful in reducing morbidity and mortality in endemic areas. However, the genotype I (GI) JEV has replaced the genotype III (GIII) as the dominant circulating strain in Asia, reducing the efficacy of current vaccine strains derived from GIII [19,20,21,22,23,24,25]. With the most successful vaccines available, JEV still causes about ten thousand deaths annually around the world [26]. Novel safe and efficacious vaccines and antivirals against flaviviruses are urgently needed. JEV vaccine strain SA14-14-2 is one of the most successful vaccines ever developed, with over 3 billion doses administered in China [27,28]. The attenuation mechanism of the JEV vaccine strain SA14-14-2 has been intensively studied and partly elucidated [29]. As a safe and efficacious vaccine, the JEV vaccine strain SA14-14-2 has been used as a backbone for constructing vaccine candidates against ZIKV, YFV, TBEV, and duck Tembusu virus [30,31,32,33]. This vaccine strain is a potential backbone vector for the development of vaccine candidates against other pathogenic flaviviruses. Thus, we aim to de novo synthesize this virus and establish a rapid recovery method for flaviviruses.

Flavivirus chimeras based on this vaccine strain backbone have been explored extensively as vaccine candidates for their safety properties [34,35,36,37,38,39]. One of the commercial JEV vaccines was a chimera based on the YFV 17D vaccine strain backbone, expressing prME of JEV [40,41]. ISFs have a safety advantage as backbones for constructing chimeric flavivirus vaccine candidates due to their inability to infect mammals. CYV, first isolated in Chaoyang, Liaoning province of China in 2008, is a dISF that infects only mosquitoes but not mammals [42]. Although CYV does not cause diseases in humans and animals, the easy manipulation of its genome should facilitate an understanding of the flavivirus host restriction mechanism and phylogenesis, as well as the development of countermeasures against pathogenic flaviviruses. Since the ZIKV outbreak in 2016 in Brazil, many efforts have been devoted to the development of safe and efficacious vaccines against ZIKV. Arthropod-borne flaviviruses including ZIKV are difficult to control because of their persistence in inaccessible wildlife hosts. A chimeric ZIKV vaccine candidate based on a CYV backbone expressing prME of African lineage ZIKV strain MR766 was developed as a proof of concept to control ZIKV in wildlife hosts [43]. 

CxFV, first isolated in Japan in 2007, and subsequently in Africa, the Americas, and Europe, is widespread in tropical and subtropical regions [44,45,46,47,48,49,50]. Unlike CYV, CxFV phylogenetically belongs to cISFs, which are not grouped with mosquito/vertebrate flaviviruses. The phylogenetical relationship between cISFs and dISFs remains incompletely understood. The mechanism behind the inability of dISFs to infect vertebrates has been intensively studied and partly elucidated [36,51,52]. However, little was known about the determinants of the host specificity of CxFV. In addition, the effect of a CxFV infection of mosquitoes on their ability to transmit mosquito/vertebrate flaviviruses and the evolutionary origin of the narrow host tropism of CxFV remain unclear. A CxFV reporter virus should facilitate our understanding of cISFs, as demonstrated with dISFs [53].

Reverse genetics has been a useful tool for understanding the biological properties and pathogenesis of flaviviruses. Conventional infectious clone construction is time-consuming and difficult due to the toxicity of the flavivirus genome to *E. coli* [7,33,54,55]. CPER is a PCR-based infectious clone assembly technique that does not demand the construction of plasmids harboring full-length cDNA of the viral genome and the transformation of *E. coli* [56]. In contrast to the construction of full-length infectious clones in various plasmids, CPER significantly reduces workload and increases feasibility in viral reverse genetics. CPER has been used to rescue as large a genome as SARS-CoV-2 and other positive strand RNA viruses [57,58,59]. Herein, using CPER, we de novo synthesized the JEV vaccine strain SA14-14-2 in days. With some technical modifications, we also successfully applied the CPER technique to generate a chimeric virus based on the CYV backbone by replacing its prME with that of a contemporary ZIKV and a Culex flavivirus reporter virus expressing GFP. The CPER-based flavivirus de novo synthesis system should help develop countermeasures against pathogenic flaviviruses and understand flavivirus host interaction.

## 2. Materials and Methods

### 2.1. Cells, Viruses, and Plasmids

African green monkey kidney Vero cells (ATCC, CCL-81) and baby hamster kidney BHK-21 cells (ATCC, CCL-10) were maintained at 37 °C, 5% CO_2_ in a high-glucose Dulbecco’s modified Eagle’s medium (DMEM) that was supplemented with 10% fetal bovine serum (FBS, Gibco, Waltham, MA, USA), 10 mM 4-(2-hydroxyethyl)-1-piperazineethanesulfonic acid (HEPES), and 1% Penicillin Streptomycin (P/S, Gibco, Waltham, MA, USA). The Aedes albopictus C6/36 cells (ATCC, CRL-1660) were maintained in RPMI 1640 Medium (Gibco, Waltham, MA, USA) supplemented with 10% FBS, 10 mM HEPES, and 1% P/S at 28 °C and 5% CO_2_. ZIKV GZ01 strain (accession number KU820898) was kindly provided by Prof Cheng-Feng Qin from the Academy of Military Medical Sciences. Viral genomes and DNA linkers were de novo synthesized as DNA fragments and cloned into the pUC57 vector by Tsingke Biotech. 

### 2.2. CPER, Transfections, and Virus Recovery

JEV. JEV strain SA-14-14-2 genome sequence (GenBank accession number D90195) was used in this study. The whole viral genome was divided into four segments, which were chemically synthesized and provided by Tsingke Biotech. A synonymous mutation (G2291A) was introduced into the viral genome to eliminate a type IIS *SapI* restriction site as a genetic marker. A linker DNA fragment comprising cytomegalovirus (CMV) promoter, the first 75 nt and last 118 nt of the JEV genome, the hepatitis delta virus ribozyme sequence (HDVr), and the simian virus 40 polyA signal was also purchased from Tsingke Biotech. Four JEV cDNA fragments were amplified from cDNA plasmids using high-fidelity Primestar MAX (TaKaRa, Kusatsu-shi, Japan) and corresponding pairs of primers (Table S1). All the primers used in this study were prepared and stored at a concentration of 10 μM. The final concentration of primers in PCR reactions was 0.4 μM. PCR products were analyzed using gel electrophoresis and purified using DNA cleanup (Thermo Scientific, Waltham, MA, USA). Purified JEV cDNA fragments and the linker DNA fragment were mixed in equimolar amounts (0.1 pmol each) in a 50 μL reaction containing 25 μL of Q5 High-Fidelity 2X master mix (New England Biolabs, Ipswich, MA, USA). The following cycling conditions, as recommended by Yin Xiang Setoh et al. 2017, were used: initial denaturation at 98 °C for 30 s, followed by 12 cycles of denaturation at 98 °C for 10 s, annealing at 55 °C for 20 s, and extension at 68 °C for 6 min, followed by a final extension at 68 °C for 10 min [60]. The CPER products (5 μL and 10 μL) were then transfected directly into confluent Vero cells grown in a well in 12-well plates with 2 μL Lipofectamine 2000 (Life Technologies), in accordance with the manufacturer’s instructions. When cytopathic effect (CPE) was observed at 7 days after transfection, the culture supernatants were harvested. 

ChinZIKV. The CYV genome sequence (GenBank accession number NC_017086) was used in this study. The whole viral genome was divided into four segments, which were chemically synthesized and provided by Tsingke Biotech. The genome of ChinZIKV was designed by replacing the prME gene of CYV with that of ZIKV. The complete genome sequence of ChinZIKV is provided as supplementary note1. To prepare DNA fragments used for construction of ChinZIKV, four DNA fragments were amplified from CYV cDNA plasmids by PCR using high-fidelity Primestar MAX. The prME fragment was reverse transcribed from the ZIKV genome using PrimeScript II High Fidelity One Step RT-PCR Kit. Primers used for amplifying DNA fragments were designed to ensure 49–82 bp overlaps between adjacent fragments (Table S1). A linker fragment comprising 49/73 bp terminal sequences overlapping the UTRs of the CYV genome, the OpIE2-CA promoter, the HDVr, and the polyA signal was designed and chemically synthesized by Tsingke Biotech. OpIE2-CA is a truncated and optimized promoter, derived from Orgyia pseudotsugata multicapsid nucleopolyhedrosis virus and has a higher transcription efficiency than the CMV promoter in mosquito cells [52]. The CPER method for ChinZIKV was the same as that used for JEV. The CPER products (5 μL) for ChinZIKV were transfected directly into confluent C6/36 cells grown in a well in 12-well plates with 2 μL Lipofectamine 3000 (Life Technologies), in accordance with the manufacturer’s instructions. Instead of Lipofectamine 2000, Lipofectamine 3000 was used for transfection of ChinZIKV CPER product to achieve higher transfection efficiency in C6/36 cells. CPE in infected cell cultures was monitored daily and supernatants were harvested when CPE was observed at 7 days after transfection.

CxFV-GFP. The CxFV genome sequence (GenBank accession number AB377213) was used in this study. The whole viral genome was divided into two fragments and were chemically synthesized, provided as plasmids by Tsingke Biotech. The genome of CxFV-GFP was designed by inserting the GFP gene cassette into the capsid gene. The complete genome sequence of CxFV-GFP is provided as supplementary note2. A DNA fragment comprising GFP, self-cleaving foot-and-mouth disease virus 2A peptide (FMDV-2A), and mutated first 34 amino acid of capsid (C34) coding sequence was also chemically synthesized, provided by Tsingke Biotech. The genome of CxFV circularizes by base-pairing between the capsid gene and 3′ end of the viral genome in the course of virus replication [61]. The second C34 was synonymously mutated to avoid its interference with the base-pairing between the first C34 and the 3′ end of the viral genome (supplementary note2). In total, six DNA fragments with 32–86 bp overlaps between adjacent fragments were amplified from corresponding plasmids and were assembled into a single circular DNA by CPER as described for JEV. The CPER products (5 μL) for CxFV-GFP were transfected directly into confluent C6/36 cells grown in a well in 12-well plates with 2 μL Lipofectamine 3000, in accordance with the manufacturer’s instructions. Green fluorescent signal was monitored daily and was observed at 4 days post transfection. Supernatants were harvested at 7 days post transfection and recovered CxFV-GFP was further propagated to prepare viral stock.

### 2.3. Viral RNA Extraction, RT-PCR Amplification, and Sanger Sequencing

For JEV, CxFV-GFP, and ChinZIKV, viral RNA was extracted using PureLink RNA Mini kit (Thermo Fisher Scientific, Waltham, MA, USA) from the culture supernatant of infected Vero or C6/36 cells according to the manufacturer’s instruction. RT-PCR was performed to amplify viral genomic fragments with viral RNA as template using PrimeScript II High Fidelity One Step RT-PCR Kit (TaKaRa, Maebashi, Japan). The RT-PCR reaction mixture (50 μL) contained 25 μL of 2X One Step High Fidelity Buffer, 2 μL of each primer (10 μM), 1 μL of PrimeScript II RT Enzyme Mix, 4 μL of PrimeSTAR GXL for 1 step RT-PCR, 1 μL of viral RNA sample, and 15 μL of RNase Free dH_2_O. Assays were performed using T100 Thermal Cycler (Biorad, Hercules, CA, USA) under the following cycling conditions: 45 °C for 10 min, 94 °C for 2 min, 30 cycles of 10 s at 98 °C, 15 s at 55 °C, and 30 s at 68 °C. The primers used are described in Table S1. The resulting DNA fragments were sequenced by Sanger sequencing at TsingKe Biotech. The prME coding sequence of ChinZIKV was sequenced after 6 passages in C6/36 cells. DNA fragments containing GFP region were reverse transcribed from serially passaged CxFV-GFP and were analyzed using gel electrophoresis.

### 2.4. Indirect Immunofluorescence Assay

Confluent Vero, BHK-21, or C6/36 cells grown in 48-well plates were infected with ZIKV or ChinZIKV at a multiplicity of infection (MOI) of 1. At 3-day post infection, cells were fixed and permeabilized with pre-cold acetone–methanol mixture (1:1) for 10 min. After 1 h blocking with 1% bovine serum albumin (BSA) diluted in PBS at 37 °C, cells were incubated with a mouse monoclonal antibody (1:500 diluted) (GeneTex, Cat No. GTX57154) against the flavivirus envelope protein for 1 h at 37 °C. After three washes with PBS, the cells were incubated with goat anti-mouse IgG conjugated with Alexa Fluor 488 (1:500 diluted) (ThermoFisher Scientific) for 1 h at 37 °C. Cell nuclei were stained with DAPI (Solarbio, Beijing, China) for 10 minutes. Fluorescence images were acquired using the Zeiss Axio Observer inverted microscope armed with a ×20 objectives.

### 2.5. Cytopathic Effect Analysis and Fluorescence/Bright Field Microscopy

For JEV, 100 μL supernatant of transfected culture was inoculated onto Vero monolayer grown in a 12-well plate and washed once in PBS after 1 h incubation at 37 °C and 5% CO_2_. Infected Vero cells were maintained in DMEM with 2% FBS, 10 mM HEPES, and 1% P/S. For ZIKV, confluent Vero cells grown in 12-well plates were infected with ZIKV at an MOI of 1. Cells were washed once in PBS after 1 h incubation at 37 °C, 5% CO_2_ and were maintained in DMEM with 2% FBS, 10 mM HEPES, 1% P/S at 37 °C, and 5% CO_2_. Confluent C6/36 cells grown in 12-well plates were infected with ZIKV or ChinZIKV at an MOI of 1. Cells were washed once in PBS after 1 h incubation at 28 °C and 5% CO_2_ and were maintained in RPMI 1640 with 2% FBS, 10 mM HEPES, 1% P/S at 28 °C, and 5% CO_2_. CPE in infected cells was monitored daily and imaged by Zeiss Oberserver A1 microscope.

### 2.6. Plaque Morphology for JEV

JEV from viral stock was tenfold serially diluted in DMEM with 2% FBS, 10 mM HEPES, 1% P/S, inoculated onto Vero monolayers grown in 6-well plates, and incubated at 37 °C for 1 h. After incubation, supernatants were replaced with 2 mL overlay medium (DMEM containing 2% FBS, 10 mM HEPES, 1% P/S, and 1% low melting point agarose) and cells were incubated at 37 °C, 5% CO_2_ for 4 days. Cells were fixed in 4% formaldehyde for 30 min and overlay medium was removed. Cells were stained with 0.1% crystal violet for 30 min and imaged.

### 2.7. Viral Growth Kinetics for JEV and ChinZIKV

Confluent Vero cells grown in 12-well plates were infected with JEV at an MOI of 0.1 for 1 h, washed three times with PBS, maintained in DMEM with 2% FBS, 10 mM HEPES, 1% P/S at 37 °C, and 5% CO_2_. Culture supernatants were harvested daily for 4 days successively. Confluent C6/36 cells grown in 12-well plates were infected with ChinZIKV at an MOI of 0.1 for 1 h, washed three times with PBS, maintained in DMEM with 2% FBS, 10 mM HEPES, 1% P/S at 28 °C, and 5% CO_2_. Culture supernatants were harvested daily for 5 days successively. Viral RNA in culture supernatants was assessed using real-time qPCR using Quantstudio 3 (Applied Biosystems, Waltham, MA, USA), M5 HiPer Direct Viral RNA qPCR kit (Mei5 Biotec, Beijing, China), taqman probes, and primers (Table S1). Viral RNA samples were prepared according to the manufacturer’s instructions. The M5 mixture (final volume: 25 μL) contained 18 μL of buffer, 1 μL of each primer (10 μM), 1 μL of specific probe (10 μM), 1 μL of enzyme mix, and 3 μL of viral RNA sample. Real time PCR was performed under the following cycling conditions: 50 °C for 10 min, 95 °C for 3 min, 40 cycles of 10 s at 95 °C, and 30 s of annealing/extension at 60 °C. Viral RNA levels were determined from standard curves. Viral RNA standards are extracted from the culture supernatant and were quantified by digital RT-PCR using AccuMini (ZHENZHUN BIO, Shanghai, China).

## 3. Results

### 3.1. Development of CPER for JEV Vaccine Strain SA14-14-2

**Figure 1:**
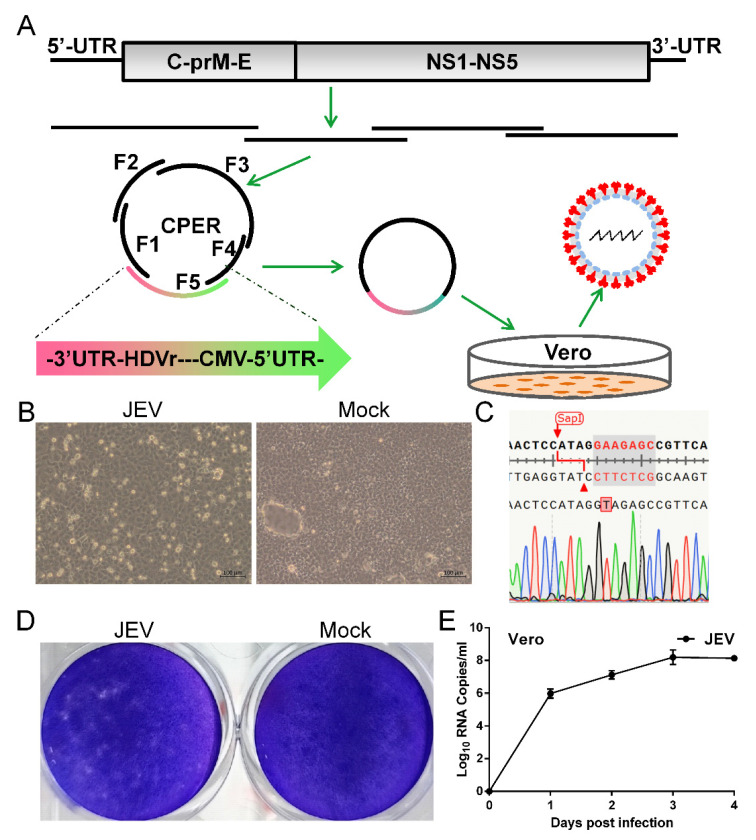
**Development of CPER for JEV in Vero cells.** (**A**) Scheme for assembly of JEV genome using CPER and virus recovery. (**B**) CPE caused by recovered JEV in Vero cells and mock-infected Vero cells was included as negative control. Vero cells were infected culture supernatant from transfected cells (passage 0 JEV) and CPE was imaged at 3 days post infection (dpi). (**C**) Confirmation of successful generation of JEV by sequencing of the genetic marker. RT-PCR was performed on viral RNA extracted from passage 3 culture supernatant. Elimination of an *SapI* restriction site by an A-T synonymous mutation was identified as the designed genetic marker using Sanger sequencing on the resulting DNA fragments. (**D**) Plaque morphology of JEV in Vero cells. Confluent Vero cells infected with JEV were fixed with 4% formaldehyde at 5 dpi and stained with 0.1% crystal violet. (**E**) Growth kinetics of JEV in Vero cells. Cells were infected with JEV at an MOI of 0.1. Culture supernatants were harvested at the indicated time points. Viral RNA levels in the supernatants were determined using real-time PCR. Data represent an average of two experiments and error bars indicate the standard deviation.

To generate JEV vaccine strain SA14-14-2, we designed a construction strategy similar to that used for ZIKV and SARS-CoV-2 [57,58,60]. A CMV promoter was added upstream of the complete viral genome, which was followed by the HDVr and the polyA signal. The CMV initiates viral RNA transcription using cellular machinery and the HDVr ensures an authentic viral 3′ end (Figure 1A). A silent mutation (G-A) was introduced into the viral genome at position 2291 to eliminate a type IIS *SapI* restriction site as a genetic marker. The whole construct was divided into five fragments, ranging from 1.3 to 3.2 kb in length, with 47–100 base pair (bp) overlaps between adjacent fragments. The linker DNA fragment used to circularize the whole construct contains the CMV promoter, the HDVr, the polyA signal, the first 75 nt, and the last 118 nt of the viral genome (Figure 1A). A total of five DNA fragments (each 0.1 pmol) were assembled into a circular product in a single CPER (50 μL). Two different volumes of the CPER product (5 μL and 10 μL) were transfected into Vero cells independently. Both the experiments successfully rescued JEV evidenced by significant CPE in Vero cells infected with JEV (5 μL) (Figure 1B). Vero cells infected with JEV shrank and became rounded and detached from the glass, whereas mock-transfected cells firmly adhered to the glass. After three passages in the Vero cells, elimination of the type IIS *SapI* restriction site at position 2291 as the genetic marker was confirmed by Sanger sequencing performed on reverse transcribed cDNA of the viral genome (Figure 1C). This genetic marker of synonymous mutation distinguishes the rescued JEV and the parental virus without causing phenotypic change. Then, we assessed the plaque morphology of JEV in the Vero cells. Plaques were formed in cells infected with JEV whereas the mock-infected cell monolayer remained intact (Figure 1D). Next, we evaluated the replication kinetics of JEV in the Vero cells which were used for the production of the JEV vaccine [62]. JEV replicated efficiently and the viral genome in the culture supernatant reached 10^8^ copies/mL at 3 dpi (Figure 1E). Together, these data demonstrate that the JEV vaccine strain SA14-14-2 is de novo synthesized in days using the CPER method, without the time-consuming construction of a full-length infectious clone. 

### 3.2. Generation of Chimeric ChinZIKV by CPER in Mosquito Cells

**Figure 2:**
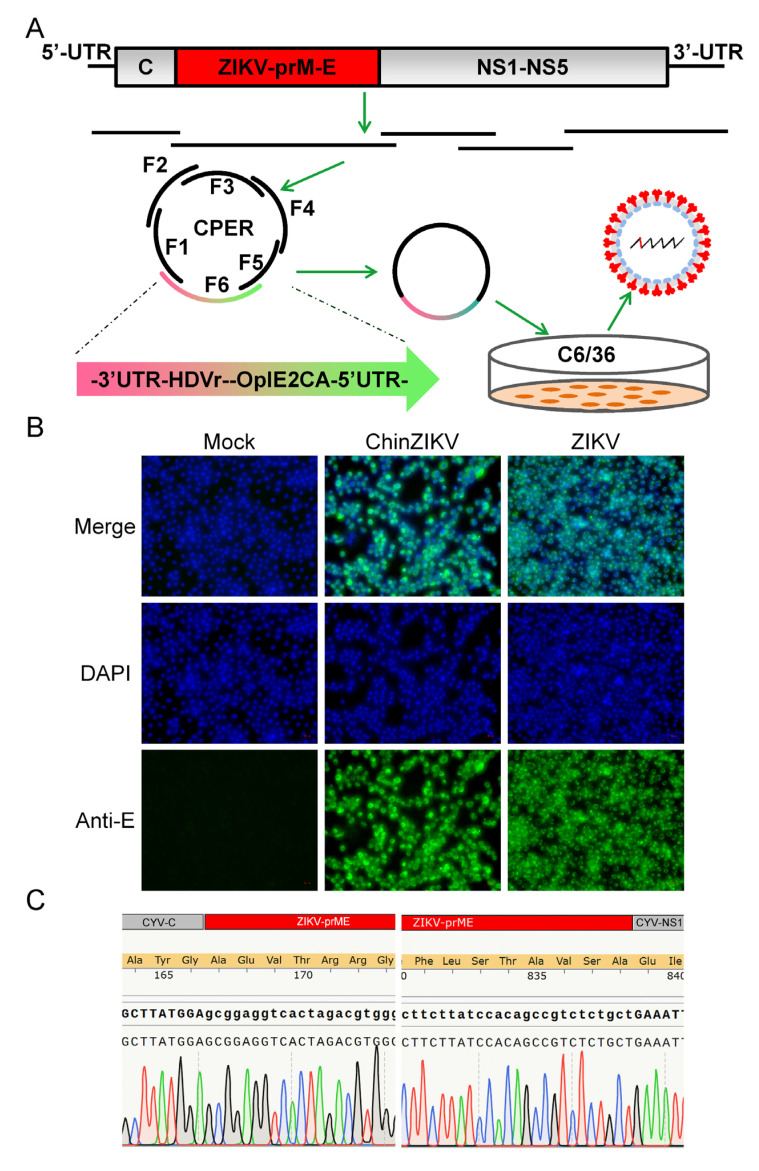
**Generation of ChinZIKV by CPER in C6/36 cells.** (**A**) Scheme for assembly of ChinZIKV genome using CPER and recovery. (**B**) Confirmation of generation of ChinZIKV using IFA. E protein expressed by ChinZIKV was identified using IFA analysis with a monoclonal antibody against Flavivirus E protein (green). C6/36 cells infected with ChinZIKV were fixed and immunolabeled at 5 dpi. Cell nuclei were stained with DAPI (blue). ZIKV was included as a positive control. (**C**) Confirmation of generation of ChinZIKV using Sanger sequencing. RT-PCR was performed on ChinZIKV genome to amplify ZIKV-prME region. The resulting DNA fragments were analyzed using Sanger sequencing.

To construct a chimeric ChinZIKV virus, the CYV genome sequence was obtained from NCBI. The construction strategy used for ChinZIKV recovery was similar to that used for JEV with some modifications. Four DNA fragments spanning the ChinZIKV genome were amplified from corresponding plasmids except that the prME fragment was amplified from ZIKV genome RNA. A linker fragment comprising the OpIE2-CA promoter, the HDVr, the polyA signal, the first 41 nt, and the last 65 nt of the CYV genome, as well as a spacer sequence of 2913 bp in length, was used to circularize the construct. A total of six DNA fragments were assembled into a circular product in a single CPER (Figure 2A). CPER product (5 μL) was directly transfected into C6/36 cells, without any intermediate purification procedures. ChinZIKV was successfully recovered, supported by CPE in transfected C6/36 cells. Supernatant from the transfected cell culture was harvested when CPE was observed at 7 days after transfection. To further confirm the successful generation of ChinZIKV, harvested supernatant was inoculated onto fresh C6/36 cells and IFA was performed. Like ZIKV, ChinZIKV expressed ZIKV E protein, identified by an anti-flavivirus E protein monoclonal antibody (Figure 2B). In addition, Sanger sequencing performed on viral RNA extracted from the supernatant showed that the prME gene sequence of ChinZIKV was identical to that of ZIKV (Figure 2C). The sequence at two ends of the ZIKV prME gene and flanking regions was as designed. Taken together, using the CPER methodology, we successfully generated a chimeric ChinZIKV in mosquito C6/36 cells.

**Figure 3:**
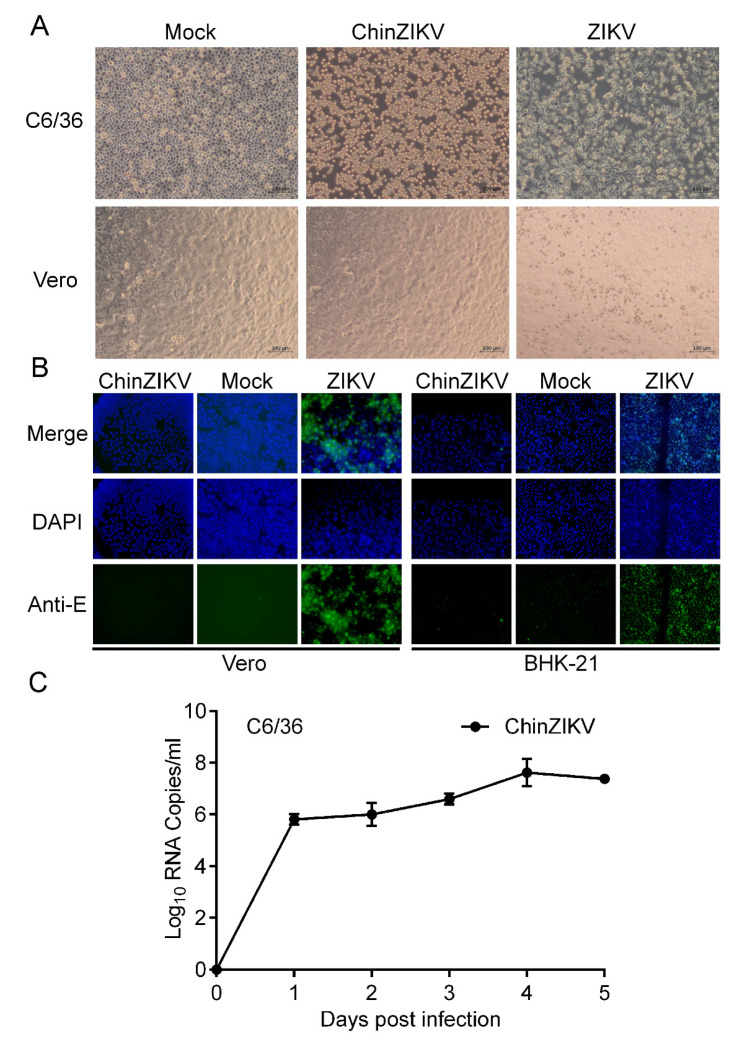
**ChinZIKV fails to infect vertebrate cells.** (**A**) ChinZIKV infected C6/36 cells and caused significant CPE but not Vero cells. As a control, ZIKV infected and caused significant CPE in both cells. Confluent C6/36 and Vero cells were inoculated with ChinZIKV or ZIKV at an MOI of 1. CPE was monitored daily and imaged at 5 dpi. (**B**) Confirmation of ChinZIKV’s inability to replicate in Vero and BHK-21 cells by IFA. ChinZIKV was inoculated onto Vero and BHK-21 cells and IFA was performed on both cells with the monoclonal antibody against Flavivirus E protein (green) at 5 dpi. Cell nuclei were stained with DAPI (blue). No ZIKV E protein was detected in cells inoculated with ChinZIKV. ZIKV was included as a positive control. (**C**) Growth kinetics of ChinZIKV in C6/36 cells. Cells were infected at an MOI of 0.1. Supernatants were harvested at the indicated time points. Viral RNA levels in the supernatants were determined using real-time PCR. Error bars represent the standard deviation from two independent replicates.

We further characterized ChinZIKV in mosquito as well as vertebrate cells. ChinZIKV was inoculated onto Vero cells to determine its cell tropism. ChinZIKV caused no morphological changes in Vero cells, whereas ZIKV-infected cells exhibited detachment and shrinking, as well as rounding, suggesting the inability of ChinZIKV to infect vertebrate cells. In contrast, both ChinZIKV and ZIKV infected and caused significant CPE in C6/36 cells (Figure 3A). To further define the host tropism of ChinZIKV, we performed IFA in Vero cells to detect ZIKV E protein expressed by ChinZIKV. Unlike ZIKV, no ZIKV E antigen was detected in Vero cells inoculated with ChinZIKV, demonstrating that ChinZIKV fails to replicate in Vero cells. Similar results were obtained when IFA was repeated on another vertebrate cell line BHK-21 cells (Figure 3B). To evaluate the genetic stability of ChinZIKV, we next serially passaged ChinZIKV six times in C6/36 cells and the Sanger sequencing of the prME gene revealed only a synonymous mutation (T2298C) in the E gene. The Analysis of replication kinetics showed that ChinZIKV replicated efficiently in C6/36 cells and the viral genome in the culture supernatants reached 10^8^ copies/mL at 4 dpi (Figure 3C). Collectively, these results demonstrated a live ChinZIKV expressing functional ZIKV prME proteins was successfully generated using the CPER method.

### 3.3. Utilizing CPER for Generation of a Reporter CxFV in Mosquito Cells

**Figure 4:**
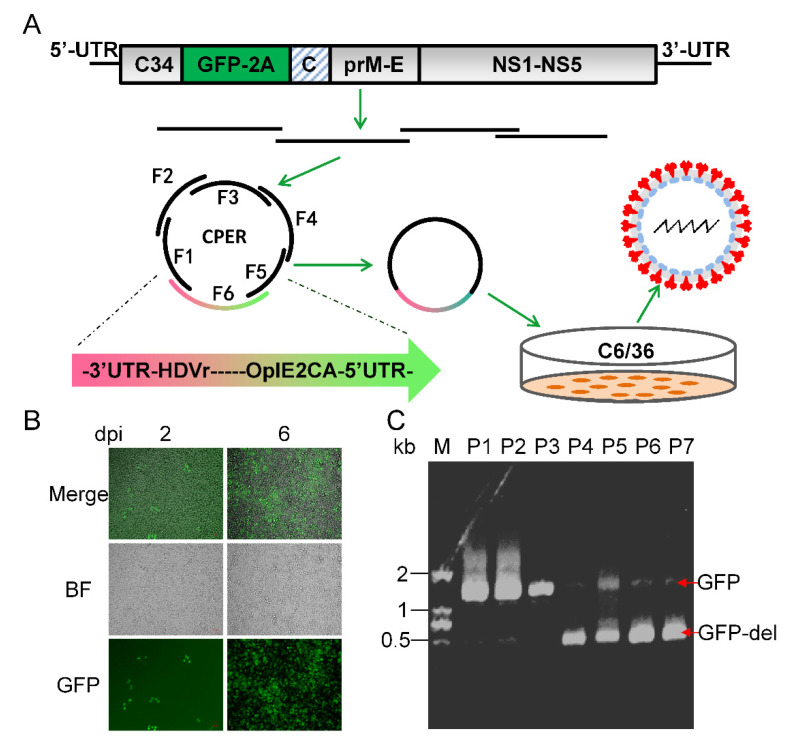
**Generation of CxFV-GFP using CPER and its genetic stability.** (**A**) Schematic diagram of design and construction of CxFV-GFP. The first 34 amino acid of capsid (C34) was duplicated and placed upstream of GFP. A self-cleaving FMDV 2A peptide was inserted downstream of GFP, followed by a codon scrambled C34 sequence (represented by slanted lines). (**B**) Detection of GFP in C6/36 cells infected with CxFV-GFP using fluorescence microscopy. (**C**) Genetic stability of CxFV-GFP. RT-PCR was performed on CxFV-GFP RNA extracted from serially passaged viruses to amplify the fragment containing the GFP gene. The fragment is 1045 bp in length and GFP is 720 bp. CxFV-GFP was passaged in C6/36 cells at an MOI of 0.1. Culture supernatant from each passage was harvested at 4 dpi.

To construct a reporter CxFV expressing GFP, we designed the genome of CxFV-GFP as previously described for the JEV reporter virus [63]. As shown in Figure 4A, C34 was duplicated and placed before GFP to ensure the circularization of the viral genome during replication as it contains cis-acting elements [8,9]. C34 is also responsible for the initiation of the translation of the entire ORF using cellular machinery. The self-cleaving FMDV 2A peptide was inserted downstream of the GFP to ensure proper cleavage between the GFP and the downstream polyprotein [64]. A second C34 sequence was synonymously mutated to disrupt its cyclization sequence to avoid interference with viral genome cyclization.

We adopted a construction and recovery procedure for CxFV-GFP similar to that of ChinZIKV. The whole genome of CxFV-GFP was divided into six fragments with 32–86 bp overlaps between adjacent fragments. We codon-optimized the GFP gene for better expression in mosquito cells. Notably, the shortest fragment was as short as 98 bp. A total of six fragments were assembled into a single circular CPER product. A fluorescent signal was observed in infected C6/36 cells as early as 2 dpi and increased during the course of infection, suggesting the successful recovery of CxFV-GFP (Figure 4B). Reporter viruses harboring foreign genes have been reported unstable and are prone to loss of the reporter gene during passaging [65,66]. To test the genetic stability of CxFV-GFP, we serially passaged CxFV-GFP in cell culture and monitored the magnitude of fluorescence in the infected cells. CxFV-GFP began to lose its reporter gene after three passages, which was corroborated using gel electrophoresis analysis of RT-PCR products containing the GFP gene (Figure 4C). Thus, CxFV-GFP is not genetically stable. Taken together, using the CPER method, we have de novo synthesized a live reporter CxFV-GFP.

## 4. Discussion

Reverse genetics has played a very important role in the understanding of flavivirus pathogenesis and the development of countermeasures against pathogenic flaviviruses [9,35,54,60,67,68,69,70]. However, the conventional construction of a full-length infectious clone and transformation of *E. coli* hosts is difficult and time consuming due to the large size and toxicity of virus genomes [7]. Although the usage of a low copy plasmid, in vitro ligation, a special *E. coli* host, a bacterial artificial chromosome (BAC), or the insertion of an intron, to some extent, reduces the difficulty of *E. coli*-dependent methods, bacterium-free CPER totally eliminates the step of constructing a full-length infectious cDNA clone and the transformation of *E. coli* hosts [7,55,70,71,72,73]. CPER is a rapid and simple method of constructing recombinant plus strand RNA viruses. We have successfully applied this method to de novo synthesize wild type and recombinant flaviviruses in mammalian cells as well as mosquito cells. As a testament to the simplicity and rapidness of the CPER method, we and other research groups successfully rescued plus-strand RNA viruses in days [57,58,59,60]. Combined with the chemical synthesis of DNA, CEPR methodology enables researchers to obtain infectious viruses in days without the transport of infectious virus samples, especially contagious and dangerous ones. This is very helpful in the case of an emerging pandemic, such as COVID-19, because most laboratories do not have access to virus samples in the early stages of an outbreak and virus isolation from various clinical samples is technically demanding [74]. 

Theoretically, viruses rescued using the CPER methodology are not monoclonal due to mutations introduced by polymerase. The mutations are mainly introduced in the preparation of DNA fragments using PCR because the CPER per se does not amplify DNA as PCR would. In theory, the CPER only doubles the amount of DNA by bridging the gaps between the annealed fragments [60]. Thus, most mutations can be avoided through the preparation of DNA fragments from the digestion of plasmids instead of PCR [75]. Moreover, the virus generated using the CPER method can be further purified through plaque purification [63]. To some extent, even viruses recovered from an infectious clone plasmid extracted from *E. coli* are a virus mutant swarm called a quasispecies due to the low fidelity of viral RNA-dependent RNA polymerase [75]. Infectious subgenomic amplicons (ISA) methodology is another bacterium-free virus recovery technique that directly transfects a mixture of DNA fragments spanning the full-length virus genome into permissive cells to rescue infectious viruses [76]. This method presumably exploits cellular recombinase to construct a linear full-length virus genome. Circular heterologous DNA is more resistant to cellular exonuclease digestion than DNA fragments, and is therefore more stable than linear DNA in cells. Thus, CPER is theoretically more efficient than ISA for the recovery of viruses.

Many chimeric flaviviruses have been constructed and evaluated as vaccine candidates [31,32,33,34,35,36,37,38,39,77]. As a safe and efficacious vaccine, the JEV vaccine strain SA14-14-2 is also a potential vector for constructing chimeric flaviviruses vaccine candidates. Since GI JEV has replaced GIII JEV as the dominant genotype in Asia, it will be promising to construct a chimeric JEV by replacing the prME of the JEV vaccine strain SA14-14-2 with that of a contemporary GI JEV. The mechanism underling dISFs’ inability to infect vertebrate cells have been partly elucidated. Dong-gang virus, a member of dISFs that is phylogenetically close to CYV, is not able to replicate in vertebrates because its motifs in UTRs fail to interact with host proteins [78]. Similarly, the ChinZIKV generated in this study is unable to infect vertebrate cells due to its’ parental CYV, consistent with findings in other chimeric flaviviruses based on an ISF backbone [42,79,80]. Few research efforts have been focused on cISFs such as CxFV partly because they are not genetically relevant to pathogenic flaviviruses such as dISFs. However, we suggest that chimeric flavivirus vaccines based on dISFs are worth exploration because, in theory, they are less likely to infect vertebrates. Reporter viruses have been widely used in the field of virology [53,65,81,82,83]. We constructed a reporter CxFV expressing GFP with the newly developed CPER method to facilitate relevant research. Notably, a DNA fragment as short as 98 bp, with an overlap as long as 100 bp, as well as two doses of CPER product were used in the generation of viruses in this study, indicating that the CPER methodology is of high plasticity.

## 5. Conclusions

In summary, we developed reverse genetics systems based on CPER methodology for both mosquito/vertebrate flaviviruses and insect specific flaviviruses. The viruses recovered in our study are potential vectors for the development of prophylactic and diagnostic tools to combat flavivirus outbreaks. Combined with synthetic DNA, emergent flaviviruses can be de novo synthesized in days, even in the absence of virus samples, bypassing time-consuming construction of full-length infectious clones for viral genomes toxic to *E. coli* hosts. The PCR-based reverse genetics described here is rapid and simple, providing a useful tool for research on flaviviruses and other potential plus-strand RNA viruses. 

## Data Availability

The data presented in this study are contained within this article.

## Supplementary Materials

The following supporting information can be downloaded at: https://www.mdpi.com/article/10.3390/vaccines11071250/s1, Table S1: Primers and probes used in the study; Supplementary note1: The complete sequence of ChinZIKV; Supplementary note2: The complete sequence of CxFV-GFP.
