# Supplementary material for: Rapid Generation of Recombinant Flaviviruses Using Circular Polymerase Extension Reaction
Source: Vaccines (Basel). 2023 Jul 17;11(7):1250. doi: 10.3390/vaccines11071250 (PMC10383701; doi:10.3390/vaccines11071250)
Supplement: Supplementary file 1 [file vaccines-11-01250-s001.zip › vaccines-2444880-supplementary.pdf]

Supplementary information

Rapid generation of recombinant flaviviruses using circular polymerase extension reaction

Hao-long Dong [1†], Mei-juan He [1†], Qing-yang Wang [1], Jia-zhen Cui [1], Zhi-li Chen [1], Xiang-hua Xiong [1], Lian-cheng Zhang [1], Hao Cheng [1], Guo-qing Xiong [2], Ao Hu [2], Yuan-yuan Lu [2], Chun-lin Cheng [3], Zhi-xin Meng [3], Chen Zhu [1], Guang Zhao [1], Gang Liu [1*] and Hui-peng Chen [1*]

1 Academy of Military Medical Sciences, Beijing 100071, China

2 Institutes of Physical Science and Information Technology, Anhui University, Hefei 230000, China

3 School of Life Science, Hebei University, Baoding 071000, China

* Correspondence: jueliu@sohu.com (G. Liu), huipengchen@yeah.net (H. Chen)

† These authors contributed equally.

Table of Contents

Supplementary table1: Primers and probes used in the study.

Supplementary note1: The complete sequence of ChinZIKV.

Supplementary note2: The complete sequence of CxFV-GFP.

Table S1: Primers and probes used in the study

Primers used for the amplification of JEV fragments	1F	ATCTGTGTGAACTTCTTGGCTT
	1R	AACACTTGGTGAACGGCTCTACCTATGGAGTTGAAGACCCCTCC
	2F	GAGGGGTCTTCAACTCCATAGGTAGAGCCGTTCACCAAGTGTTTGG
	2R	ATGCGGTCTTCTTTCACACT
	3F	TTTCCACACACTATGGCACAC
	3R	ATTATGGCCTCTCTCCGGTA
	4F	CAGGACGCTAGGGGAGCAGT
	4R	CACCACCAGCTACATACTTCG
	Linker-F	CAAACAGCATATTGACACCTG
	Linker-R	AACTGTTTAAACTGCATTAATCTCTCGATTCTTC
Probe and primers used for the real time PCR of JEV	F	AGAGCACCAAGGGAATGAAATAGT
	R	AATAGGTTGTAGTTGGGCACTCTG
	Probe	CCACGCCACTCGACCCATAGACTG
Primers used for the amplification of ChinZIKV fragments	1F	CTATAAATACAGCCCGCAACGATCTGGTAAACGAAAAGTATATTCTACGTGTGC
	1R	CATATAGTATGCACTCCCACGTCTAGTGACCTCCGCTCCATAAGCCATACAACTC
	2F	TGAGTTGTATGGCTTATGGAGCGGAGGTCACTAGACG
	2R	TCTCTGTGTGTTATGTCAAGGCTGCATCCAATTTCAGCAGAGACGGCTGTGGATAAG
	3F	TTGATCTTCTTATCCACAGCCGTCTCTGCTGAAATTGGATGCAGCCTTGACATAAC
	3R	AATACTACAGGGGTAATGTTCTCACAACCTGCTGACTGCGCGATCGC
	4F	AACGACAGCACCTACGCTAGTGCGATCGCGCAGTC
	4R	TAAATATCCTTTATCTACCAGCCATCTGAGCTTTGCTGAACCCCGGGATAC
	5F	AAAGAGTGGAAATGTAACAGGAGGTCACGCTGTATCCCGGGGTTCAG
	5R	ACAACCTCCAAACTACCTTT
	Linker-F	CACCAGACTACACGGCACA
	Linker-R	ACCAACTATGTTACAGAATGCAC
Probe and primers	F	ATGTATGATGCAGAACACCCCTA

used for the real time PCR of ChinZIKV	R	GTGTTAGCTCCTTGACCACTC
	probe	FAM-5'ATCCATGGTAGGTCCACGTCT3'-BHQ1
Primers used for the amplification of CxFV-GFP fragments	1F	ACGCCTGGTATCTTTATAGTCC
	1R	CGCCCTTGCTCACCATCTTCTTTTTCTCCTCCTTCCTTCC
	2F	GGAAGGAAGGAGGAGAAAAAGAAGATGGTGAGCAAGGGCG
	2R	GGAACTTGTACAGCTCGTCCATG
	3F	GTCCTGCTGGAGTTCGTGACC
	3R	CTGCGCACCGCCGGAGACC
	4F	GCACATCTCAGCCGGTCTC
	4R	ACACTCTACTGTTGTCCGTCA
	5F	GCCCACAACACTTCCGTCTG
	5R	CATCTTTTACTTTCACCAGCGTT
	Linker-F	GTAAAAGATGACGCCTGGTATCTTTATAGTCCTGTCGGGTTTCGCCACCACTGATTTGA
	Linker-R	TACCAGGCGTCATCTTTTACTTTCACCAGCGTTTCTGGGTGAGCAAAAACAGGAAGGCA
Primers for the amplification of the GFP region in CxFV-GFP	GFP-F	TCTCGCAGAAGCGGGAAAGGACG
	GFP-R	CTGCGCACCGCCGGAGACC

Supplementary note1:

The complete genome of the ChinZIKV.

Red: ZIKV-prME

gaaaagtatattctacgtgtgcattctgtaacatagttggttggagaagtattttggatataacacgtttgaataggtttattgtgaatcgaaataccg
atggcgaataaacccaagaagcccggaagacgggctatcgatatagtgagacgtgcgttgcctcgcgtctcgggaccgaaaaaggtcttaa
agaaggcaacacaaacagtcatggaaagcttggctggcatacgggcaaccgttgcctacctgctctacatgacattcctcggcaacaaggtc
agcaaggccacgagagctaagtttcgcagtgcgaaaaagagtgatctgataaagatcctcagtagcttcaagagaacggttacaaaccttctg
gctagtgtgcaaaagaggaagagaaaggcgaagagatcggtcacgacgcccagcatggctttgattctattgacaatgagttgtatggcttat
ggaGCGGAGGTCACTAGACGTGGGAGTGCATACTATATGTACTTGGACAGAAACGATGC
TGGGGAGGCCATATCTTTTCCAACCACATTGGGGATGAATAAGTGTTATATACAGATCAT
GGATCTTGGACACATGTGTGATGCCACCATGAGCTATGAATGCCCTATGCTGGATGAGG
GGGTGGAACCAGATGACGTCGATTGTTGGTGCAACACGACGTCAACTTGGGTTGTGTA
CGGAACCTGCCATCACAAAAAAGGTGAAGCACGGAGATCTAGAAGAGCTGTGACGCT
CCCTTCCCATTCCACTAGGAAGCTGCAAACGCGGTCGCAAACCTGGTTGGAATCAAGA
GAATACACAAAGCACTTGATTAGAGTCGAAAATTGGATATTCAGGAACCCTGGCTTCG
CGTTAGCAGCAGCTGCTATCGCTTGGCTTTTGGGAAGCTCAACGAGCCAAAAAGTCAT
ATACTTGGTCATGATACTGCTGATTGCCCCGGCATACAGCATCAGGTGCATAGGAGTCA
GCAATAGGGACTTTGTGGAAGGTATGTCAGGTGGGACTTGGGTTGATGTTGTCTTGGA
ACATGGAGGTTGTGTCACCGTAATGGCACAGGACAAACCGACTGTCGACATAGAGCTG
GTTACAACAACAGTCAGCAACATGGCGGAGGTAAGATCCTACTGCTATGAGGCATCAA
TATCAGACATGGCTTCGGACAGCCGCTGCCCAACACAAGGTGAAGCCTACCTTGACAA
GCAATCAGACACTCAATATGTCTGCAAAAGAACGTTAGTGGACAGAGGCTGGGGAAAT
GGATGTGGACTTTTTGGCAAAGGGAGCCTGGTGACATGCGCTAAGTTTGCATGCTCCA
AGAAAATGACCGGGAAAAGCATCCAGCCAGAGAATCTGGAGTACCGGATAATGCTGTC
AGTTCATGGCTCCCAGCACAGTGGGATGATCGTTAATGACACAGGACATGAAACTGAT
GAGAATAGAGCGAAAGTTGAGATAACGCCCAATTCACCAAGAGCCGAAGCCACCCTG
GGGGGTTTTGGAAGCCTAGGACTTGATTGTGAACCGAGGACAGGCCTTGACTTTTCAG
ATTTGTATTACTTGACTATGAATAACAAGCACTGGTTGGTTCACAAGGAGTGGTTCCAC
GACATTCCATTACCTTGGCACGCTGGGGCAGACACCGGAACTCCACACTGGAACAACA
AAGAAGCACTGGTAGAGTTCAAGGACGCACATGCCAAAAGGCAAACTGTCGTGGTTC
TAGGGAGTCAAGAAGGAGCAGTTCACACGGCCCTTGCTGGAGCTCTGGAGGCTGAGA
TGGATGGAGCAAAGGGAAGGCTGTCCTCTGGCCACTTGAAATGTCGCCTGAAAATGG
ATAAACTTAGATTGAAGGGCGTGTCATACTCCTTGTGTACTGCAGCGTTCACATTCACC
AAGATCCCGGCTGAAACACTGCACGGGACAGTCACAGTGGAGGTACAGTACGCAGGG

ACAGATGGACCTTGCAAGGTTCCAGCTCAGATGGCGGTGGACATGCAAACTCTGACCC
CAGTTGGGAGGTTGATAACCGCTAACCCCGTAATCACTGAAAGCACTGAGAACTCTAA
GATGATGCTGGAACTTGATCCACCATTTGGGGACTCTTACATTGTCATAGGAGTCGGGG
AGAAGAAGATCACCCACCACTGGCACAGGAGTGGCAGCACCATTGGAAAAGCATTTG
AAGCCACTGTGAGAGGTGCCAAGAGAATGGCAGTCTTGGGAGACACAGCCTGGGACT
TTGGATCAGTTGGAGGCGCTCTCAACTCATTGGGCAAGGGCATCCATCAAATTTTTGGA
GCAGCTTTCAAATCATTGTTTGGAGGAATGTCCTGGTTCTCACAAATTCTCATTGGAAC
GTTGCTGATGTGGTTGGGTCTGAACACAAAGAATGGATCTATTTCCCTTATGTGCTTGG
CCTTAGGGGGAGTGTTGATCTTCTTATCCACAGCCGTCTCTGCTgaaattggatgcagccttgacataa
cacacagagaaatcaagtgtggagatggcattttcatcttcagggatgcagggggctggagagacaaatatgtctttcatcctggctctccaaa
aaccctagctgcagctatttggaaaggatggaatgatggaatctgtggagttaggtcagccacccgcatggaacatgaaatgtggaaacaaat
tgaaaatgagctcaatgggattctagaagaaaatgacatcaaattgagtgtcgttgtgaagaatgccaatggcacatatccacgaggaaccaa
gtcattaacccgaaacacaacaggactgcagtacggatggaagtcgtggggcaagacaatgtttgtttcagtgccaatagccgagaacacttt
cataatcgatggaaatgatgaaggagagtgtgcctcggacaaacgcgcttggaatacatttaagatagaggagtttggcacaggaataatgaa
aaccaaagtcttcctggatttggctgacgcacaaacagaatactgcgacacggaactgttgggagctgctgtgaaaggcaacaagtctgttca
tggcgaccccggattgtggatgactgcttctaaggaatcaggcgactggaaactggaatcattgtcaatgacggagagcaggcggtgcctgt
ggcccgattcgcacaccatttggggaagaggagtgctggagtcaaaactcatcttaccatcaatgttcggaggaccagtttctcacatgaacac
cagacctggatatgccacgcaattgtcaggaccctggaacaatgtcccactggatgttgtgtttgaggagtgtcctggtactaaagttgtggtgg
aaaacaattgcacgaacagaggagaatccatcagatctaccacagatagtggcaaaatcattccagaatggtgctgccggaaatgcaccatg
ccacccctcacttaccgcacgccagatggatgctggtatgccatggaaatcagaccaaaaaaggcaagcgaagaaagtctgttgcgatcaaa
ggtttcagctggaacatttcaaggaattgatgatttttccttagggctcctagtgctaatcattttcgtgcaggaaggtcttaaaagaagaatgacc
agccggtacataatgttggctgcactaggactattgctggcagcagtgttgggtgacctcacatacaatgacatagcaaggtacgtgatcatgg
ttggtgtagcatttgcagaaatgaacaacgggggagacttgattcatttagccctgattgccacattcaaagtgcaacctggttatctgcttttcttc
cttcttcgcaaacaatggtcaccacgggaaagtaccattttggcttcagcagcagtcgttctgcaaatttgtgcggctgcatggcaatcaacaaa
gtctatgcaagtgctgaatgctcttgctatgggatggctgtacatcagagccattgtggtacccggagctctttccaaagcaatgcccctcatttg
catgtgcgtcccaggcgtgttgagcctgacgccacatgctataagagtcagcatggttacaatagcggcaggcactctcatcaaaggcacga
agggaacatcagttcggaagcacatgccctatttttaggactagtaggagccgtggccggactggatccattaggtatgcttggatactcctta
ttaacgtacagcagcggaaagaggtcatggccagcaggagagattatgacggcagtcggtctaacatgcgctatgatcggggcattaagcg
gaaacgcgatgaatgatattgctgggcctgcggcagctgcctctctgatttttgtggcttacgcaataagtgggagatctgcagacgtgttcttg
gaaaaggctggtgaaatttcatggattgacgacgcagcagtttctggatcaagtccaagggtggacgtgcaagttaccgacgggggggatttt
cggcttcgccacgaagccgaggcttcgtggctaaagaatggagtgatggctttctgtctcgttctggctggagtacacccgttagcgattccgg
tagcggggttgatctggttcggtttcgtaaaaagtgggaggcgtggcacggttctctgggatataccacatccgattgcatcatctgccccaagt
gtggaggacggatgttacagagtgatgtcaagaaggctcattggttctacgcaagtaggagtgggagtgatgaaagactcagtgtttcacaca
atgtggcacgtcacccgtggcgcctctctaacgagtggtaacggtagaatggatccatattgggcggatgttcgagaagatctcatcagctac
ggtgggccatggaagctcaacagcacttgggatggatcttctgaagtgcagttaatagcagtgcaaccccagaaaatacccgagaacgttca

aacaactccaggaaggttcgtcatgtcagacggaactgaggttggggctgtggtgcttgactacccgtcaggcacttcaggttccccaatcgt
ggacaaagatggaaatgtggttggactgtatgggaatggtgtcatgcttaacgacagcacctacgctagtgcgatcgcgcagtcagcaggttg
tgagaacattacccctgtagtattccacccggacatgttgaaaaagggaaagctgagcgttatggacctacatccaggagcgggaaaaaccc
gcaaggtgttgccacaggttctcaaagaagcagtggccaagaagttgaaaacccttgtactggccccgacgcgtgttgtagctaaggaaatg
cacggagctctagctggattgccggtgagataccaaacctcggccgttgaaaccaaagggagtggaaatgagctgatagacgtcatgtgtca
tgcgacatttacgtacagacaattgacaccaggaagaatggttaattaccaattgtatgtaatggatgaggcacatttcactgacccagcttcaat
tgcggcaagaggaataatcgcaacacgggtcaagctaggagaagcagctgcgattttcatgacggcaacacctccaggaactcttgatgcat
tcccagaatccaatagccatattgaagatgaggaaagggaaattccagacaaggcatggagcaccggattcgaatggattacagattacaca
ggcaaaactgtgtggtttgttccatcaattcgaactgggaacatcatagcgagctgcctcgcgaggacagggaagaaatgcgttgtgttgaata
gcaaaacattcaatgacgagtttccaaagaccaagagtggaacgtgggactttgtgatcacaacggacatatctgaaatgggagcaaatttca
aagcctcacgagtcattgactgtagaacgtcaatcaaaccaacattggcctatgtcccttctgagagagtagtgttgggatctccaaaacccata
agcccagctagtgcagcacagcgtagagggagagtgggtcgagaccccactcagcttggtgaccagtacatttacggaggcgaggttggt
gacgacttctccaacatggtgcactggacagaagcgaagatcttaatggacaacatcatggtaccaggcggtctgtacccacagttctacgag
ccggaggctgacatgctaagtgaaacggatggacacttcaggctggatatgaccaaacgtgacgtattcaaggatttggtgcgcaaggcaga
cttacccatatggctggcttatcaggtggccaagcatggtcatgaatacaaagacagaagctggtgccatagtgggccatctggacatctcatc
tacgatgattacggacagacagttgaatacaagcttgtgaatggcgagaggaagatactgcaaccaagatggattgaccaacgcacatacca
ggagaaaacagcactcaaggctttcatcgaatttgctgagggacgccgaagttatgttcctataatagaagtgctcggcaaacttccacagcat
ttcgccgacaaaactattgatgcggctgacaccttcaagactgtgcttacagccactccaggaagtagagcatacaggcttgcagttgacaac
ctacccgatgcagcagagacggcaatattcgtgacgatggtagggttcatgaccatgggaatactaattttcctgatggcacccaaaggcatg
acacgcatgtcgctgggattcatgaccatcatggcagctacgtattttctgtgggcttcaggcatggctggatatcaaatagcggcaatgcaatt
agtggctttcattctcttcgtagtcttggttccagaaccaggatcgcagagatcagtacaagacaatacgattgccatggtcctaattggaatact
aagcattgcagcattgattgcggcaaatgagacaggactgctggagaagacaaaaaatgatttcgtgtctttgttaccggttaaagccccgaac
aacaatccctggaagtttgattttagcgtggacttgagaccggcgacatcatgggctttgtatgtagtgattgctacaatgcttgggcccattttgg
agcatgcgattgtgactcacttcacaagtatatcaattgcagccatcacgaatcaagcaggaatactgctatcaatggataaaggaacgccattt
ttcaacttggactggggcgtcattctgttgggagtggggtgttggtctagcataacggggacaaccttgttgtgtgcgatcggactctcctgcgtt
cacttctcaatgactctacctggagtcagggctaaagcagcgagagaagctcaaaacagaaccgcagctggagtatcaaagaaccctattgt
ggatggagtcaacactataaacgtcacagcgcccccaggaatggaccccatgtatgagcgcaaacttggactgtggatgctcctgatcatgg
cggtcatatccacagcggtaaacagagacttgactcacttgatggaactaggcatactgggaagcgcagcacttggcccactcatagaaggg
aactcttccacaatctggaacacctcagtggcatcttcactgtgcaacttgatgagaggacagtatctagctggaatacctctgacatacacgct
ggtcagaaacctctcgctaaaaggggttccccgtagaggagtcaccataagcaacacccgagggatggaatggaaacgcaaactaaacgc
aatgaacaaggaaacctttcagagatacagacgagatgggatagttgaggtggacagaacagcagcgcgggaagctttaaagagtggaaat
gtaacaggaggtcacgctgtatcccggggttcagcaaagctcagatggctggtagataaaggatatttacgcctgatgggggatgtcacaga
cttgggatgcgggcgaggtggatggtgttattacgcagcagctcaaagaaacgttgtatctgtacgaggcttcaccaaaggaggtgacggac
acgaagaacccattccggtgcagacttatggatggaacattgtcactctgcgtagcaaagtggacgttttctacatgcctacacacatgacaga
cactctattgtgtgacattggagagtcatcgtctaacatgctagtagaagaagagaggaccctacgcgtgctgaatctggttgaagagtggatc

agaaagagtaagccaactcacttttgttgcaaggttcttgctccatacatgccaaatgtattggagaagatagacaagctaatgaaagttcatgg
aggtgctttagtgagagtgccactctcccgcaacagcacacatgagatgtattgggtatctgatgcaagaggtaatgccatgaatgcagtggc
atctctgagtagaacacttttggacaggatgatacacttgacaggaaaagtgcactgggaagatgatgtcaacctgggaactgggactagagc
agtgaacagtgtcgctgatccaccaaactgggacaaaattggactccgagtgagaaaacttgaacaagaatacaaagcttcatggatgtatga
tgcagaacacccctacaagacgtggacctaccatggatcatatgagacttccacatctggaagtgcttcatcaatgataaatggagtggtcaag
gagctaacacacccatgggacacaaactccggagtgacaaacgtttgcatgacagacacaaccccttttgggcaacagcgcgttttcaaaga
aaaggtcgacaccaaagcaatggaacctccaactggaaccagagaagtgatgaggattgtgaacagatggttgacaaactacctgtctagaa
caaagaagcctagactgtgtactccagatgagtttattgctaaggtgaactctgatgcggctctgggaacaatgttcaatgaccaagggaactg
gccaagcgcaaaagatgcagtgagagaccatggattctggaggcaagtcgacattgagcgacaacaccacttagagggaagatgcgtctc
atgtgtctacaacatgatgggaaagcgtgaaaagaaattgacggaatttggaagggccaagggcagcagagcaatctggtacatgtggctag
gagcaaggtacttggaatttgaagccctaggattcctgaacgaagatcattggctctcgagagagaattccaaaggtggggtcgaagggattg
gattacaatacctaggctacatccttgaggatatgggaggaatgacaggaggccaaatgtatgcagatgacacagccggctgggacaccaa
gataacgaatgctgacctagaggatgagatggagatcacaaagctgatggcacacacccacaaaaaattggccacggccattatggatctca
cgtacatgaacaaggttgtgagagtgatgaggccaggcaagggaggaaagacgctaatggacatcattagcaggaaggaccagagaggt
agcggacaggtcgtgacttacccccctcaacacgtggacaaacctcaaggtgcagttgattaggatggctgaaggcgaaggagtgatcctacc
tgaagacactctgtcactcaccggctgcagtagacgcaatttagaaatgtggctggtgaggaacggagaagaacgattaacccggatcgctg
ctagcggagatgatgtggtggttaagcccattgatgaccgatttgcagaagctctgcactttctcaacagcatggccaagatcagaaaagatat
caatgaatggaagccctcaacaggctggaacagttgggagggtgtgccgttttgttcacaccatttccacaagctcagcttaaaggatggaag
aacactcacagttccatgcagagatcaggacgaacttattggaagagctagagtgtctcctggagcagggtggacgttgcgagaaaccgca
ggactcagtaaggcgtatgcgcaaatgtggatgctgatccacttccacagaagggatctacgaaccatcgcgttcgcaatttgcagtgcagtg
cctaaagactgggttcccacaggaaggacatcgtggtccatccatgctcgaggtgaatggatgacgaatgaagacatgttggcggtgtggaa
tcgagtgtggatcactgagaatccatacatgttcaacaaagaaacaattcacgactggcgagatgttccatacctgcggaaacaactggacaa
aaattgcggatcaatgataggagtgaggtccagagccacgtgggctgaaaacattcgtgtttcagtgaaccaggtacgtggttacattggtaaa
catgagaactacctcgattacctccaagcccagaatagattcagcattcctgccgaattcaccatgggcaacatattgatctaagacactgaga
gaaaagactgaaaaacaattgacgctgagagtcaggcctaaatgccaccggatgatagtagacggtgctgcctgcagctatcacatataaact
ggcgccttatatgtttattagccgacaggggggacacatgcacccagcagcccgagctggacaaggcatgtgtactagcggttagaggagac
ccccccaaaaatgaagggcactatatcgacacttgggaaagaccagaggtactcgctgattcaccgccaccagactacacggcacagcgc
gccggaaaaggtagtttggaggttgtaaaacaacaagtatct

Supplementary note2:

The complete sequence of the CxFV-GFP.

Red: C34; Green: GFP; Blue: FMDV-2A; Purple: Mutated C34

agtttttaaaaacttcggcttggttacaccgcagattggttacacctacacaaggcttgagttgtttataatagtcgtttttctcgcagaaATGGG
AAAGGACGACGGTAAGAAGAAGAAGGGGCCGGGCTCCTCCGGATGGTTGTTACCACC
GGGGCGCGCTGGCTTAGGAAGGAAGGAGGAGAAAAAGAAGatggtgagcaagggcgaggagctgt
tcaccggggtggtgcccatcctggtcgagctggacggcgacgtaaacggccacaagttcagcgtgtccggcgagggcgagggcgatgcc
acctacggcaagctgaccctgaagttcatctgcaccaccggcaagctgcccgtgccctggcccaccctcgtgaccaccctgacctacggcgt
gcagtgcttcagccgctaccccgaccacatgaagcagcacgacttcttcaagtccgccatgcccgaaggctacgtccaggagcgcaccatct
tcttcaaggacgacggcaactacaagacccgcgccgaggtgaagttcgagggcgacaccctggtgaaccgcatcgagctgaagggcatc
gacttcaaggaggacggcaacatcctggggcacaagctggagtacaactacaacagccacaacgtctatatcatggccgacaagcagaag
aacggcatcaaggtgaacttcaagatccgccacaacatcgaggacggcagcgtgcagctcgccgaccactaccagcagaacacccccatc
ggcgacggccccgtgctgctgcccgacaaccactacctgagcacccagtccgccctgagcaaagaccccaacgagaagcgcgatcacat
ggtcctgctggagttcgtgaccgccgccgggatcactctcggcatggacgagctgtacaagttcCTGTTGAATTTTGACCTT
CTTAAGCTTGCGGGAGACGTCGAGTCCAACCCTGGCCCCATGGGAAAAGATGATGGA
AAAAAGAAAAAAGGACCAGGAAGCAGCGGATGGTTATTGCCACCAGGAAGAGCAGG
ATTGGGACGAAAAGAAGAGAAAAAGAAAaaggagaagaggggtgtgcggagcacatctcagccggtctccgg
cggtgcgcagcaccgccgcggaggcggaacaggtcctagggccggaggtctactaggacgtttgggagttggctggggatcgattctcca
ggaggacattgtgcacgcactcatgcacttagtgctggtgctacactctgttttcatcgcaattgatcgacgcttgcgctcactgacacggcgcg
tgactgctcttgaggctaaacgctctgcgaagaacgctgtacggatcgcattcatgctcaccggcgtgatgatggtgctgggcgccgtcgtcat
tgatatgcaggtctccacggtgaaagggacacagatcttcgaggggaagaccaaccgcactgatcatgtgcacttgtttaagctccccacgga
tggctgctggagtggcacattggttatgaaaaagtgccccaaggtcgctgatcttgcgaaggatctggaaggagtggattgtggttcatcatgg
acggaattcactttgcgctaccatcgatgcgtgccaaagaagcgtgaacgacgagttgccagcaccaaccagaagctagattttctggcgga
agtggaactggtgacgttcaaagctataagggagaacaaaaccatcgctatcatcgtcttgttgtgcgtggctattgcgaagagatggccgatg
tgggtggtgatactactcgcaattggaacgtggactacggtgaagggagaatttgttgaaccattgtacacactaaaagcggaaaagatgacc
atgttgcagacaataatgcgaccggacgaaagctacgtgatttccacgccgaacggacttcttgagtttcgcaccggagcagccgagatatat
ggcggccaatggatgcgtgaattgctcgctgattgtcacgtgaacgcgtcattctcgactgacgtgtgtcctggaggatctcagctgaacatgg
gtgagatcaatggtaaagaacgtgtttgctcaacccagccctacaatcgtggatggggaactggatgcttcaagtggggaatcgggtttgttgc
cacgtgtgttgagctgcattgcggagaggggtataacgtgtcgtcaatcgctaggtcatccatcgtgatgaacatcacagcgaaattccactca
gtggatgacgtccagcaactcattagtgatgttcctgtgacttttcgatttgcaaagctgggcaatgccgctgttacttgtcggctagaatcagag
cgattgcttcttgactactatcacgtgacaggcaaacaccacgaaggactgtttgtgcggtctcaaatcgatgagtggcccggagcgcacgcc
actgctagtggaagggccggtttggaacggattgtggtctggggagacgccagggctaatgaagttttggtgaaaaacattttggaaccccag
ctcgtctgggataatgccattgccacgcaggacggttttcgtgatgttggctattcttgtcagatcatgcttgacaaactggttagcggtagttttaa

ggactgcccggggatcaagtcttcagtcttcgtccaaagcggtttcggatacgacggagtggtgatgacaagcttgtccaaagcaactaatga
gagctgctctgtggggctgacgtgccacggatgctacctccttgcgacgaagatggtgttcggacctggaacttcgaccgcaagggcatttgt
tgggtgtgggaatcacaccggaacccttgtggttggaggaacgacaatccacgttgagtgtgtgttgaacccgatttcgcaaggttggagaat
ggccaagcatgtggttgacaagtaccggaggtttggaactgctggagtgggaggcgtgtggcatgactttgttggaaagttttcattgttcagc
ctgttgtctagctcgacgattctcgtcggactcgccgctctggtgtttcttgacaaacgaattgtgttcttgctacttttgtgcggatactttgtctaca
ccaaggcagatgtgggttgcggatttgaccctgagagaaaggttgtgtcctgcggatcaggagggttcgtctggaaaagcttgtcacaatggc
cgacccgggaacattcagttgaactcgatgaccaacacttgatcacggctcttgttacggaacagttgaagaagacgaacaaggtgtgtatcat
ttgtgaggacgtcttgcaatgcgccgccgctcgtagtgctgttggggagattacccacgttgacaatgagattgtttacgttaacacttcgttgtct
tttgacaggacttttcctcgaattccgaagaaggttcatggcgtgaaaattggtgacttaaccatgcagctagccctagcttcagttgggggagc
ggttgacgccagccaatatggtgagttgagcagcgggttcttgagccgcaccaagatcgcggaaaccagtgaacacaaggtcatccgtgtg
ataacaagtgcctctccatacgagaaaatatgtgagcaggcgttcgcgctgcagtacggtttcgtccggttcacccgcagggtgttcggttcaa
atgttgtggtcaagcccgtggcaaaaccaacagattactgccccacgtacctggccggatcgtttgtgaaaaatgacataggcgcgtacaccg
atgggatgatgtggatgaaaagcaggaaggtgaacggaacgtggacattggtcgacctggaactgacgcagagccatcaatgcatttggcc
ccaagcttatacgtttgatttgacggcctttaatgactcatcgctgttcatgccggcacaatacggcgctccgatgtctaaagcaaaccacattcc
tgggtacaagacgcaaacagagttcccttggtacaaagctgatatcgtgttgcgcgagggggtggtccccggaactcaggttgaagagagc
ccatcttgtgacaaccgcgggtccgctgtgaaagttgaccctgccattgccaagaagtggtgttgcaagacgtgcttctcagctgacaagcgg
gtatttcacttcaaggtcgacaacgattacttctaccccatggagattcgaccagcggctgttcagccagaggtgaccattgacgctgacggcg
aagacatggacgagatgacatcaatgttcgggacaatgaaggcagtcgtaccgccagtggaagggtcctatccggatttccggctcagccca
tctgtggagggagtttctcccctactggtgggcgcgcttttgcacctgctgaccattcgaaccaaacatcggtgggctcaacgcacgtgtggaa
cttggattttgttcctcctgtttggagtgccatccaacacctatgtcgggtggtcttggattggattatcgtactcgcttgctgctgttccgaatggta
cggctctgttggtgcatttctggctggcagttcagttctcatcatcgcacttgttctttttgggatgggcgctacggcaacgtgtacgatcatcagct
ggatacgcattgaccgtgttcttcgccgagtggcttctcttgaagttacggcagctgtgggaatcgacatacctcctcgaccacgtgctgttccc
gatgtacgtgatgttggcgttcaatctgaaatcacagttcgtacctgtggactcgatggtactgctgaactatgttgtgacgcatcctgcagttgc
gacggccatggtaacaggaggtgccctggtgatctattccattcgcgtgtacaagagctggggatgctctccgaacttgtggaggagtggact
tcgcgcttcgaggcgctcattagtggctggaatgtgcttggcggggttgtacgtgctctcaacgtgtctcgagttgtatcagatgccgacgacg
gcatcggtggtgttcctaggaggactactgatcgggatagtgaccaggatggctcctccagcgcacttggagttggttccggtggctggaatg
ggagtgccgttggattgcgaggaagagcccacaacacttccgtctggattagaaggaacttacggaccggatggagtggaattcacgaactt
gacggacaacagtagagtgtcaacggggttgctggtttacgtcggctgcatgggaatcatggccatcaatacctacgtcggggtcatcctgat
gtgtgcttgttggtggacaaacgcgcccgagtggctacctctctacgtggctggagtatcggtctttcgatccaatgaggtgaatgatgtcctca
tcacccctccagagtatgagcaggaagcacagctaagcaacgacttcgggcacctgcccgacggaacataccgggtggttgctcgctcgct
gttcatgaccagccacgtgggagcaggctacgccaaagacggtgtgttcaacacgctttggcatgtgacatctggtggaagcctcacatggc
aagggagaaacgtgcgcctacactctggtgatgtttaccgggacatggcttcgtatggcggatcgtggaacattgctgattccctcgaggactc
agtggttgttcgtgtcgtgcagaacgatggtagcgtactgtgtgctagaagctccacggcaagtattagcatcgatggaaagcgtgtccaggtc
attggccatgactatggaaaaggatcgtcaggatctcctgtgcacgcgttggatggtcgtgtcgttggactgtacggttatggatttttcattggat
ggaagtaccactccttgatcacaagcggagaagtcaccgcagaggaaaactttgaggaagacacagtgtctcgccggttcgttgattggcat

ccaggaaaggggaagaccagacgcgtgttggttgaagaggctaaatctcacatcgcaaaagagaaacgtctgctgatactgacgcccactc
gagttgttaaggatgaggttgtgcgagcgattgctgaggcgtgccctggagttgtggtaggatcaaacctagcgatgtaccgaaggaatgcca
tcacggtggcttgccacgcgacgctaacgcagtatctgatggagaaaggcattgacagcattcgattctccaccatcataatggacgaatgtca
cttcctggaccccctgtccatcgcctgtcgtggggtcatggattactacaatggaaaaggggttgccgtggtgtacatgagtgcgactcccccc
ggatgtgctggaaacaacggatcaaatcatcccatcgatgacgtggcgacacaatttccgcgcgaattaacagcatcgtgggtccgtgggca
agcgaatggcaagacgataatattcgttcccacacagcaccaagccaacactttggctagtgaacttggaggagtttcactgacccgcgaatc
gtttgacgttgcgatcggaaaggcccgcaagcaggagacacagttcatcgtgtcgacagacatcagcgaaatgggagcaaatctcggtgtg
cagacggtcattgacacgcgtgtggcagtgaagcctgtcattagcgaaggttccgtcatgctggagaaagttggcatcacacaggcgtcagc
catccagcgccgtggaagaacaggacgcagagaagcaggaaagtatgtctaccccattgggtctgagctggaaaacgaggccactgagtg
ggcttgttggactgaagctcaaatgattctagaccagatggcgtgcggGccgatgcgtgaggaaatcgagaacttccagccacagggcaga
tacctcttggcgcctgaaagtcgaccccggttcatcaacttcatcaagaaggatgttccagtctggcttgcgtggcactgggcgaatgcgtttga
acacaagcactcagtgctgtttcaaggacagaatgcgacgagcttgaagatcaggacggaagccggtgaccacaagtacgctccccgattc
catgacgatcgtttcgaaaagaacaacgagttggacaaacgctccaagatcatgctgtacctcaagcagcggagcaatttcaactttgacgtg
gggggcgttctgtatggactatttgtggctttccgtgacacgaacctagagcggcttggtacatcctaccgctcggcgattgagattctccatga
aatctctaacgttgatgatccaatggtttcaaacgtggtgatgggaaagtcgctacaagcttgggcggctgtcatcattgggatggtcactggga
ttgtgctactcgtcatgtttgtagtggtttgtcggatcgtgaagtggctgttcggtggaaagacaactgcacaacaaagcccccccgtactcaagc
ttcccaacgttacaaacagcaggattttgtcaattcggctcaatggtgatggctatcggaccactatgtgcggtggttgctggcattcctcccgct
ttcgtttttgttgcggtgttgggattgttcgtgataatgtgctgtagcgctaataacgtgcaccgggcttacactactgacaccgtcacgctcgttgt
catcggtgtttgcgtgtgtgtcatgggagttgttgcgtgggaaatggatctacttcccaacatccgacgggatctaggttacatcctggaaagga
tcgccgcgaggcaagagccggacatcccacaagcatctttcgcgcggccagaggttctagagttgcacatcacatctcttcctggagcgttg
gttgtgagctttgccattgctatcgttggggggggttattgttaactgtctgtccgacagcggatttctgcgcaagttgttttcgaacgaagaacagt
ccgcaacggtaatcggaggcatccagctcgctttgatttcctgggagacgatggtgccaatcgcttttgctgggttcttcgcaactacgttcgtg
acgaagatctatgggtgcatggtaggaggaatctacctggttttagctcactatgaccggaagtatgcctttacagtgaaggcgaccaaggtcc
tcatcgcgcgcaccagcaagaaagatctcgacgatgagataacaggacgagacggggtgacgcgaggccggccaaccttttacgcgttac
aaatatgttgttccctgctgtggacagtcacgtcgcctagcctgaagcatgctgtcgtgagtgtggtggtgattgtgtttgcattcctcacatttcgt
cgtcccaataacaggctgctcgtcacgtttgactattctagcgtgctgttggtcttgatgatcttcgctgaacctggtcaggtttttctggtaggtgc
tagtttactgttttggttcgtggcgcatcagtctcgcatggcgctacggtcgctggtgaagacagacgcttgcggactgggatacagatggaag
gaaatgcttaacgcgcttgacaaaaatgcgtttgacaagtaccgatcccgaggagtgaacgagacagacaagggtgatttcgtctcgcgggg
cggtttgaagatggatgagctcatcaggaagttccaatgggaaccgaagggggctgcacttgacttaggatgtggccgcggaggatggact
caacgtctggtgatggacactagggtcaactccgtgacggggttgacgctgggtggagcaaaccgggaaaatcccctaccgtttaagacga
aaggacacaacctagccgtgctaaaagccggtgttgacgtgtacgcgcttgagccgcgagactgcaacacgattgtgtgtgacataggcga
gagtgacccccggcctgaggtggagaagacaagaacgctgaaagtgctgacgatgctcgagaaatggttagtccacaatccaggtgctgc
gttctgttgtaaggtgctgtctccgtaccacctcgaagtgttgcgcaagctagagatgcttcagcacaagcacaatggaaaattggtcaggttgt
ccttgagccggaactccactgcagaaatgtactacgtctcaggtccgcgagcgaacatcgtcggctcagtttatcacgtcttaggggctctaatt
gggcgcttcaagcgtaatgacccggtgcagcaggatgctcctccaaagctcgagatgggcactcgaagtgacccccgggcaaaagtgaag

ctgcaggaccagacaatcgttgcaggtcgcgtgaagaggttgcgagaggaaaacgccagcacatggttcgtggaccgtgaacacccttacc
aatcgttcaactaccacgggtcatttgtcactgacgacatctctcccggagggcagactgtcaacccgatgatgcgccgaataatgtggccgt
gggatttcctctcccgtgtgacgacgttcatgatgacagatgtgtcaacgtacgcgcagcagaaaattctccgtgagaaggtggacacactga
cgcttgaaccagatcagcggacccgagctatcaataggttgatcatgcggcatttctctgctatgttcaaacggcgaggattggctccgagaat
cctcaccccagtggagtacatgaacaacgtgaaaagcggtgccgccattggagggtggagcaaggagatgccatggaacaaggtgcaag
aagctttggctgaccctgtcttttggcgtatggtggctgatgaacgcgctcggcacctgcgtggagattgtgagttgtgcgtgttcaacacaatg
ggcaagaaggagaagaagccgtcctctttcggcgaggcacgaggatcaaggattatctggtacatgtggctcggaagtcggtttttggagta
cgaagcacttggattcctaaacgaggaccattgggtagcgaggaagaactttccgtgcggagttggcggggtcggagtgaattactttggata
ctatctccaggaaatcatgcaaaaaggtaagtggatgattgcagatgatgttgccggatgggacacgcgcataactgaagctgacctggagg
acgagttgtggttcctgctggaccaagtgactgacccctaccacgcacagttgatacgtgttgtgttcaagttctgctacatgaacatggtcgcct
tgtttccaaggaaccatccgcagttccggagcgggacagtttttgacgttgtttcacgcacagaccaacgcggctctgggcaagtgaccacct
acgctctcaacaccgtgacgaatgggaagaaccaggttgggagaatgctggaggctgaagggctactggacgcgccgttggggatgatcg
atgggtggttaggttcccatctggaagagattttgagtggaatggtcgtggccggggatgatgtggttgtggcaaccaacaatgaaaacttcca
caccagtctaaggtacatcaccgccgcgtcaaaaacgcgcaagaacttgcaaccaaccgagccttccccgaggtacacaaactgggaacat
gttgaattctgttcccatcactaccatcctctcgtgctgcaagacggtcgtgagatcattgctccgtgtcgtgaccaacacgagatcattggccga
gcacggatccagaaagggggatcgtggacatgtcagctgccgggtgcttagcaaaggcacatgcccaaatgtgggcattgtactacttcca
ccggagggacttgcggctcggatttgccgctatcacgtcggccgtcccggtgaactggatccccacggggcggatatcgtggtcagtccac
caacacgctgaatggatgacgacacaggacatgctagaagtgtggaacaccgtgtggattgtcaacaacccatggatggcagtgaaagacc
cagtgaagacatggtcagagataccatacctcccgaagacgaaggacataaattgtggaagtttgattggtgagcgggacagggcagcgtg
gtcgaagaatatcgtggccacagttagcacaactcgacgaatcatcgagcaagaagctggaagtcagaagttcacggaagggttgcggata
cttgggcgataccgagctccaaccgacgacgtgttctggtgagaatcacgcgaatcgtagagaaccacatctctagaaaaggttaacgttgcg
aagcaacgggaaccccgtaaggaaggacaaggctgtccttgagtactaacgacactccggccccagttcccagagccagggttttagctcc
acggtgctggaagtcaccctcgcagccatggctgcacgacgcgcgcaaggaaggacatggctgtccttgggtactaacgacaccccgccc
ccagttctcaaggttagagttataacctcagggtgttggaagacatccaggccatagtagggccatcgcaagggaggattttcctcgggtactg
accataccccgaccccagtccgataggtcatggaatgaccccatggtgctgagagggcatccaaacaagctgagcatcttggattctgctccc
gtaaggaaagcgcaagctttgagcattgacaacgctccggccccagtcccccaggttatgggagaataaccccgacgtgctggaagggca
cgaatcaccgcaaggtgagggcgcacaggatagaatccaggtgactgacgccacctcccgaaatgtgtatagtaacagagcatgcctgcag
cagcaggtctccaccgttaggagacttgttgcgggcaagctcttgttcacgtct
